# The reliability of the ankle brachial index: a systematic review

**DOI:** 10.1186/s13047-019-0350-1

**Published:** 2019-08-02

**Authors:** Sarah Casey, Sean Lanting, Christopher Oldmeadow, Vivienne Chuter

**Affiliations:** 1 PO Box 127, Ourimbah, NSW 2258 Australia; 2 CReDITSS – HMRI, School of Medicine and Public Health, Newcastle, Australia

**Keywords:** Ankle brachial index, Peripheral arterial disease, Lower extremity artery disease, Repeatability, Reproducibility, Reliability

## Abstract

**Background:**

The ankle brachial index (ABI) is widely used in clinical practice as a non-invasive method to detect the presence and severity of peripheral arterial disease (PAD). Current guidelines suggest that it should be used to monitor potential progression of PAD in affected individuals. As such, it is important that the test is reliable when used for repeated measurements, by the same or different health practitioners. This systematic review aims to examine the literature to evaluate the inter- and intra-rater reliability of the ABI.

**Methods:**

A systematic search of MEDLINE, EMBASE and CINAHL Complete was conducted to 20 January 2019. Two authors independently reviewed and selected relevant studies and extracted the data. Methodological quality was determined using the Quality Appraisal of Reliability (QAREL) Checklist.

**Results:**

Fifteen studies of ABI reliability in a range of patient populations were identified as suitable for inclusion in the review: seven considered inter-rater reliability, four intra-rater reliability, and four studies evaluated both inter- and intra-rater reliability. Inter-rater reliability was found to be highly variable, with intraclass correlation coefficients (ICC’s) ranging from poor to excellent (ICC 0.42–1.00), while intra-rater also demonstrated considerable variation, with ICCs from 0.42–0.98. Meta-analysis was not possible due to the lack of statistical information reported.

**Conclusions:**

Results of included studies suggest the inter- and intra-tester reliability of the ABI is acceptable. However, inconsistencies in obtaining systolic pressure measurements, calculating ABI values, and incomplete reporting of methodologies and statistical analysis make it difficult to determine the validity of the results of included studies. Further research, with more consistent reliability methodology, statistical analysis and reporting conducted in populations at risk of PAD is needed to conclusively determine the ABI reliability.

## Introduction/background

Peripheral arterial disease (PAD) describes the process of progressive atherosclerosis affecting arteries, most frequently in the lower limb. The prevalence in the general population has been estimated at up to 19% in people over the age of 55 years [1], with incidence increasing with advancing age and in the presence of smoking, inactivity and obesity [1, 2]. The presence of PAD is associated with increased risk of mortality and morbidity from cardiac atherosclerosis [2], and, in its advanced stages, can result in lower extremity ulceration and amputation [3]. Diabetes mellitus is an independent risk factor for the development of PAD [4], and in people with diabetes, atherosclerotic plaques tend to have a more distal and diffuse distribution and there is a more aggressive disease presentation [5].

Due to the high risk of concurrent cardiovascular morbidity, mortality and lower limb complications associated with PAD, accurate and reliable diagnostic testing methods are required for screening and ongoing monitoring [2, 6]. Early detection of PAD allows for intervention and management to reduce the risk of mortality and morbidity related to atherosclerosis (lifestyle modification, pharmacotherapy, e.g. statins, antiplatelets, and measures to address systemic risk factors such as hypertension or diabetes) [7]. Current recommendations for non-invasive lower limb vascular assessment include using the ankle-brachial index (ABI) as an objective measurement of peripheral blood flow [7, 8]. The ABI represents the ratio of ankle to brachial systolic pressure and is recommended to be calculated by dividing the higher systolic pressure of the dorsalis pedis and tibialis posterior vessels at the ankle with the higher of the systolic pressures measured in the brachial artery in both arms [7, 8].

The ABI is widely used to screen for PAD in different clinical settings and by different health professionals, from general medical practitioners to specialist vascular technicians [9, 10]. Reliability of the test for accurate ongoing monitoring of lower limb vascular status has the potential to be affected by a number of factors. As an operator-dependent test, this includes the experience and skills of the clinician, particularly as multiple clinicians are frequently involved in ongoing monitoring measurements [11, 12]. There are also a number of types of equipment (e.g. automated versus manual) and methods used to measure ankle and arm blood pressures (e.g. stethoscope, Doppler, photoplethysmography probe), with variable findings as to whether the results are interchangeable [13–16]. The pre-test protocol and test environment have also been demonstrated to affect the resting ABI at measurement, with variations in body position [17], recency of tobacco smoking, caffeine intake [18, 19] and exercise [20, 21], and pre-measurement rest time [22] all likely to introduce error to the measurement and affect the test-retest reliability.

### Objectives

Given that the ABI is the recommended method for screening for the presence and progression of PAD, it is important that it is reliable. Therefore, the aim of this review was to systematically evaluate the literature to determine the inter- and intra-rater reliability of the ABI in adults.

## Methods

### Search strategy

A search of relevant biomedical journal databases from the University of Newcastle library website was performed to identify studies that consider the reliability of ABI measurement from database inception to January 2019 using MEDLINE (1946+), EMBASE (1947+), and CINAHL Complete. Truncated versions of some search terms were used to ensure that relevant studies were included (Table 1).

**Table 1:** Search terms: searches were limited to human studies

Databases: MEDLINE (1946+), EMBASE (1947+), and CINAHL Complete	
1	Ankle brachial pressure
2	Ankle arm pressure
3	Ankle brachial ind*
4	Reliab*
5	Consistenc*
6	Accura*
7	Reproduc*
8	Repeat*
9	Agreement
10	Precision
11	1 or 2 or 3 AND 4 or 5 or 6 or 7 or 8 or 9 or 10

### Inclusion and exclusion criteria

The review was conducted with reference to the Preferred Reporting Items for Systematic Review and Meta-Analysis (PRISMA) statement [23]. The following criteria had to be satisfied for inclusion in the review: published original research evaluating the reliability of the ABI in adults. Studies were excluded if the test-retest time frame made it likely that results may be affected by disease progression e.g. > 12 months. No language restrictions were applied to the database searches.

### Other sources

Hand searching of the reference list of appropriate articles was also conducted.

### Data collection and analysis

All abstracts obtained were assessed independently by SC and SL for inclusion. There were no instances of disagreement between reviewers, so arbitration by a third person (VC) was not necessary. Data extraction was performed by SC and SL. It was pre-determined that a meta-analysis of reliability outcomes for inter- and intra-rater reliability would be conducted provided there were sufficient studies that report the estimator of interest, and a measure of uncertainty for this estimator (e.g. standard error, 95% confidence interval, non-truncated *p*-value). Given the expectation for a high degree of study heterogeneity, we believed a fixed effect meta-analysis would generally not be appropriate so we aimed to only pool estimates using a random effects approach provided there were at least 5 studies [24].

### Methodological quality assessment

The studies that met the inclusion criteria were appraised for risk of bias using the Quality Appraisal of Reliability (QAREL) Checklist and qualitative methodological assessment [25]. All full-text papers were assessed for methodological quality independently by two reviewers (SC/SL), and as there were no disagreements arbitration by a third reviewer (VC) was not necessary.

## Results

**Fig. 1:**
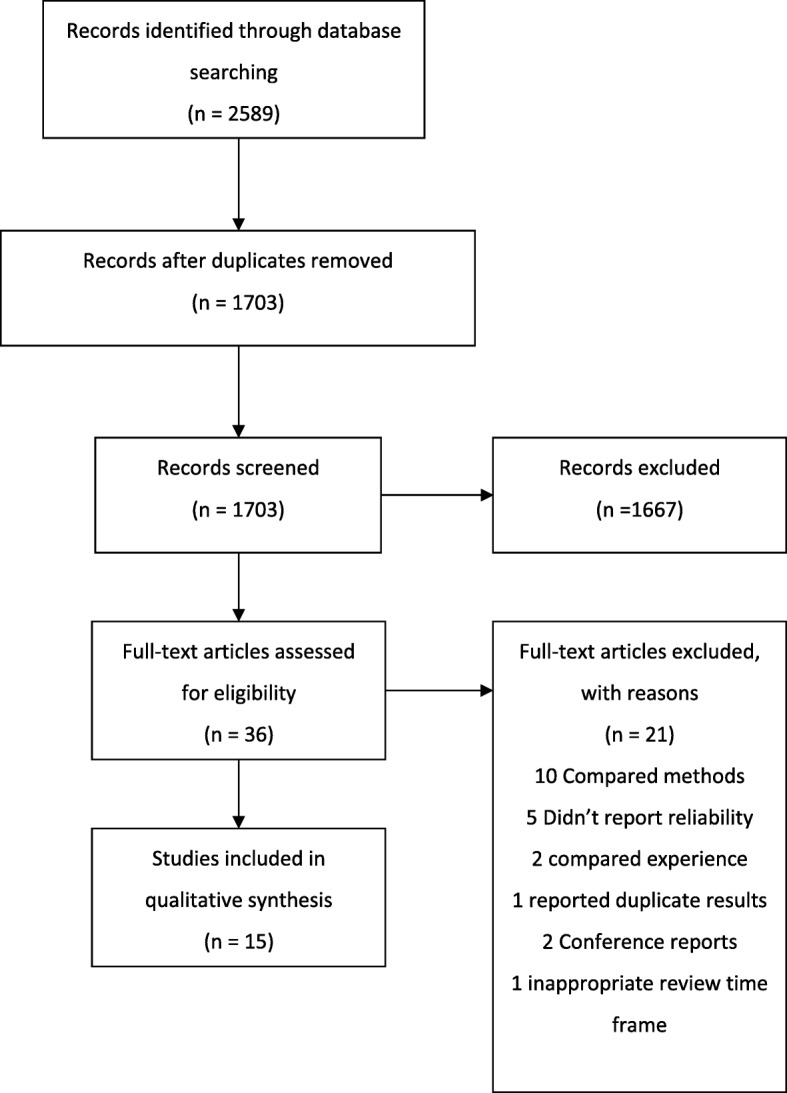
PRISMA flow chart

A total of 1703 articles were retrieved, of which 36 were identified as suitable for full-text review. Twenty-one papers were excluded based on the exclusion criteria (Fig. 1): 10 papers reported comparison of methods [26–35], five studies did not report measures of reliability [36–40], two studies compared raters’ experience [41, 42] and one reported a novel trial design, for which the reliability results were duplicated in another included paper [43]. One paper used measures repeated at up to 365 days apart, with a mean time between measures of 228 days, which is long enough to encompass changes attributable to progression of PAD [44]. Two papers were conference abstracts, for which the full text could not be obtained as the authors did not respond to a request for further information [40, 45].

Of the included papers, seven measured inter-tester reliability [12, 46–51], four assessed intra-tester reliability [52–55], and four considered both inter- and intra-tester reliability [13, 16, 56, 57].

### Characteristics and overview of included studies

The 15 studies in this review included a total of 916 participants, with data collected from a combination of one and both lower limbs (1396 limbs in total). Two studies did not state the number of limbs included [52, 53]. Eleven studies assessed inter-rater reliability [12, 13, 16, 46–50, 56, 57], and eight studies reported intra-rater reliability [13, 16, 52–57]. The characteristics of included studies are described in Table 2. Eleven studies reported participants’ gender, with more men (*n* = 416, 56.4%) overall than women, whilst gender was unreported in four studies [12, 46, 49, 50]. Most of the studies included predominantly older participants (age range (41–92 years) [12, 13, 16, 47–49, 51, 53–55, 57], however two studies recruited only younger adults (age range 22–30 years) [46, 56], one study included 18–80 year olds [52] and one study did not report participants’ ages [50]. The majority of studies [12, 47–51, 55, 57] included only participants with suspected PAD, or risk factors for atherosclerosis; three studied a mixed population including those without risk factors or clinical indicators of PAD [13, 16, 52]; two studies included only participants with diabetes [53, 54], and two studies included only healthy individuals [46, 56].

**Table 2:** Participant characteristics and reliability measure

Reference	Number (n)	Gender (M,F)	Age (years)	Height (cm)	Weight (kg)	DM & control	DM duration	Medical history	PAD	Reliability measure
Inter-rater reliability (*n* = 11)										
Aboyans et al. (2008) [13]	54	28, 26	52.8 ± 17.1	NR	NR	DM 35.2% control NR	NR	HT 38.9%, DL 38.9%, CAD 33.3% CVD 11.1% TS 22.2%,	19 IC, 25 RF, 10 healthy	ICC Doppler: Inter: 0.79 (0.70–0.85) Pulse: inter: 0.40 (0.5–0.57) Auto ABI inter: 0.44 (0.27–0.58)
Alvaro-Afonso et al. (2018) [51]	21	15, 6	67 ± 8.7	NR	NR	All DM2: control NR	NR	HT 19, DL 17, NEU 18, NEP 1, TS 6	NR	Kappa coefficient Normal: 0.4 (p < 0.001), PAD 0.7 (*p* < 0.001), MAC 0.43 (p < 0.001)
Chesbro et al. (2011) [46]	20	NR	22–30	NR	NR	NR	NR	Healthy volunteers only	None	ICC Doppler: R: 1.00 (95%CI 0.999–1.00, *p* < .001); L: 0.99 (0.997–1.00, *p* < .001)
Chesbro et al. (2013) [56]	10	5, 5	NR	NR	NR	NR	NR	‘Healthy young adults’	None	ICC Vascular Cuff Right: Trial 1: 0.853; Trial 2: 0.898 Left: Trial 1: 0.448; Trial 2: 0.938 Standard Cuff Right: Trial 1: 0.902; Trial 2:0.817 Left: Trial 1: 0.826; Trial 2: 0.867
de Graff et al. (2001) [57]	54	31, 23	66 ± 12	NR	NR	DM 36% control NR	NR	HT 43%, Dl 35%, CAD 36%, CVD 20%,	Suspected	Repeatability Coefficient/ICC Inter: day 20 / 0.92 Inter: week 27 / 0.87
Georgakarakos et al. (2013) [47]	18	12, 6	54–74	NR	NR	9 DM: 3 oral, 6 insulin	NR	HT 15, DL 9, TS 4	All PAD	Mean, Standard Error, t-test PAD: 0.77 ± 0.19, *p* = 0.95 No PAD: 1.37 ± 0.12, *p* < 0.0001 Severe PAD: 0.23 ± 0.07, *p* = 0.0002
Holland-Letz et al. (2007) [16]	108	50, 58	68.1 ± 1.5	NR	BMI 29 ± 4.3	DM 15.7% control NR	NR	HT 58.1% DL 54.8%, TS 9.2% current, 40.7% ex-, Hx vasc surg 6 subjects	68.1 ± 1.5	ICC for inter-observer: 0.423 Inter-ob SD 0.103
Jaffer et al. (2008) [50]	25	NR	NR	NR	NR	NR	NR	NR	All suspected PAD	Pearson CC *r* = 0.516 (p < 0.001)
Langen et al. (2009) [48]	20	11, 9	41–75	NR	NR	NR	NR	NR	All IC Symptoms	Interobserver variability: 10% (SD 0.8)
Mätzke et al. (2003) [12]	30	18, 15 (no of limbs)	26 > 65 yrs., 7 ≤ 65 yrs	NR	NR	8 DM, control NR	NR	NR	Ischaemic pressure lesion or rest pain	Coefficient of variation: 3.2
Span et al. (2016) [49]	136	NR	64 ± 7.8	NR	NR	19 (14%) control not reported	NR	HT66, DL 58, TS 22 current, 39 ex-	RF or IC	Coefficient of variation Doppler: 5.9% R & L legs Auto: Right 3.2% Left 3.5%
Intra-rater reliability (*n* = 8)										
Aboyans et al. (2008) [13]	54	28, 26	52.8 ± 17.1	NR	NR	DM 35.2% control NR	NR	HT 38.9%, DL 38.9%, CAD 33.3% CVD 11.1% TS 22.2%,	19 IC, 25 RF, 10 healthy	ICC Doppler: Intra: 0.89 (0.84–0.92) Pulse: Intra: 0.60 (0.44–0.73),
Chesbro et al. (2013) [56]	10	5, 5	NR	NR	NR	NR	NR	‘Healthy young adults’	None	ICC- Intra-rater Vascular Cuff Rater 1 R: 0.750; Rater 1 R: 0.696 Rater 2 R: 0.551; Rater 2 L: 0.869 Standard Cuff Rater 1 R: 0.628; Rater 1 L: 0.420 Rater 2 R: 0.620; Rater 2 L:0.585
de Graff et al. (2001) [57]	54	31, 23	66 ± 12	NR	NR	DM 36% control NR	NR	HT 43%, Dl 35%, CAD 36%, CVD 20%,	Suspected	Repeatability Coefficient/ICC Intra: day 9 / 0.98 Intra: week 22 / 0.89
Demir et al. (2016) [52]	161	87, 74	52.03 ± 18.99	165.12 ± 8.88	75.61 ± 13.4	DM NR	NR	HT 62.7%, DL 46.6%, TS 29.8%,	Mixed population	ICC Single measurement 0.808 Mean: 0.927
Faccenda et al. (1988) [53]	36	28,8	56 ± 11	NR	NR	All DM1	NR	No other hx reported	NR	Coefficient of variation 8%
Holland-Letz et al. (2007) [16]	108	50, 58	68.1 ± 1.5	NR	BMI 29 ± 4.3	DM 15.7% control NR	NR	HT 58.1% DL 54.8%, TS 9.2% current, 40.7% ex-, Hx vasc surg 6 subjects	68.1 ± 1.5	Intra-observer Variance: 0.008, SD 0.87 [0.081; 0.095]
Millen et al. (2018) [54]	66	51, 15	69.5 ± 12 yrs. (range 35–92)	NR	NR	4 DM1, 14 DM2		HT 79%, DL 68%, CAD 44%, CVD 17%, TS 15% current, 59% ex-	36 IC, 4 rest pain	Coefficient of variation: Dopplex Ability: 9.65 ± 12% Parks Flo-lab: 4.95 ± 3%
Rosenbaum et al. (2012) [55]	157	80, 77	59.1 ± 13.2	NR	NR	35 DM, control NR	NR	HT88, DL 103, CAD 14, CVD 2, TS 27 current, 49 ex-	11 PAD, all RF or IC	Coefficient of variation: Dopplex Ability: 9.65 ± 12% Parks Flo-lab: 4.95 ± 3%

*NR* Not reported, *DM* Diabetes, *HT* Hypertension, *DL* Dyslipidaemia, *CAD* Coronary artery disease, *CVD* Cerebrovascular disease, *NEU* Neuropathy, *NEP* Nephropathy, *TS* smoking, *IC* Intermittent claudication, *RF* risk factors, *PAD* Peripheral arterial disease

There was little consistency in the training and qualifications of the raters used, with experience ranging from students [46, 47] to experienced vascular technicians and/or vascular specialist doctors [12, 13, 48, 54, 57]. Six studies did not state the background of the personnel performing the test [49, 51–53, 55, 56]. The majority of the studies used Doppler and manual sphygmomanometer to measure systolic blood pressures; [12, 13, 16, 46–49, 51–53, 56] however three studies used an automated device to obtain some or all of the pressure readings [54, 55, 57] and one study did not report the method used [50]. The reported pre-measurement rest time varied from five minutes [55] to 15 min [48], with seven studies not reporting a period of rest before testing commenced [44, 47, 49, 50, 52–54]. The time between repeat testing varied from five minutes [46, 56] to 4 weeks [52]; six studies did not report time between repeated measures [12, 49–51, 54, 55]. Several different methods were used to calculate the ABI. The majority of studies [47–49, 51, 52, 56, 57] divided the highest ankle pressure by the higher brachial pressure measurement, two [13, 16] used the highest ankle pressure and the mean brachial value, and one used the lowest ankle pressure and the highest brachial pressure [55]. One study used a fully automated device that calculated the ABI value [54], and four did not state how the ABI was calculated [12, 46, 50, 53].

### Methodological quality

The quality of studies was variable with regard to reported blinding of raters, order of examination and the time between repeated measurements, with no study clearly addressing all of these variables. While most studies used appropriate statistical measures of agreement, reporting of results was frequently incomplete and the true extent of reliability could not be determined (Table 3).

**Table 3:** QAREL Checklist

Item	Aboyans et al. (2008) [13]	Alvaro-Afonso et al. (2018) [51]	Chesbro et al. (2011) [46]	Chesbro et al. (2013) [56]	de Graaff et al. 2001) [57]	Demir et al. (2016) [52]	Faccenda et al. (1988) [53]	Georgakarakos et al. (2013) [47]	Holland-Letz et al. (2007) [16]	Jaffer et al. (2008) [50]	Langen et al (2009) [48]	Matzke et al. (2003) [12]	Millen et al. (2018) [54]	Rosenbaum et al. (2012) [55]	Span et al. (2016) [49]
1. Was the test evaluated in a sample of subjects who were representative of those to whom the authors intended the results to be applied?	Y	Y	Y	Y	Y	Y	Y	Y	Y	Y	Y	Y	Y	Y	Y
2. Was the test performed by raters who were representative of those to whom the authors intended the results to be applied?	Y	U	Y	Y	Y	Y	U	Y	Y	Y	Y	Y	Y	Y	Y
3. Were raters blinded to the findings of other raters during the study?	Y	U	Y	Y	Y	NA	NA	Y	U	U	Y	Y	Y	NA	Y
4. Were raters blinded to their own prior findings of the test under evaluation?	Y	NA	U	U	U	U	Y	NA	Y	NA	NA	NA	NA	U	Y
5. Were raters blinded to the results of the reference standard for the target disorder (or variable) being evaluated?	NA	NA	NA	NA	NA	NA	NA	NA	NA	NA	NA	NA	NA	NA	NA
6. Were raters blinded to clinical information that was not intended to be provided as part of the testing procedure or study design?	U	U	U	U	U	U	U	U	U	U	U	Y	N	U	U
7. Were raters blinded to additional cues that were not part of the test?	U	U	U	U	U	U	U	U	U	U	U	U	N	U	U
8. Was the order of examination varied?	U	U	Y	Y	U	N	U	U	U	U	N	U	Y	U	U
9. Was the time interval between repeated measurements compatible with the stability (or theoretical stability) of the variable being measured?	U	Y	Y	Y	Y	Y	Y	Y	Y	U	Y	U	Y	U	U
10. Was the test applied correctly and interpreted appropriately?	N	Y	Y	Y	Y	Y	N	Y	N	U	Y	Y	Y	N	Y
11. Were appropriate statistical measures of agreement used?	Y	Y	Y	Y	Y	Y	P	Y	Y	Y	U	N	Y	Y	Y

*Y* Yes, *N* No, *U* Unclear, *P* Partly, *NA* Not applicable

### Meta-analysis

A number of the eligible papers identified lacked sufficient data relating to the main outcomes to allow for inclusion in a meta-analysis. For example, the paper by Chesbro et al., [46] provided no details on the intra-rater reliability of measurements taken using a Doppler, which was the main outcome being assessed in this review. Similarly, papers by De Graaff et al., [57] and Demir et al. [52] detailed no measure of variability for the intraclass correlation coefficients (ICC) reported, which is required when pooling results in a meta-analysis. It is not clear whether Chesbro et al. [46, 56] used data from the same population in both studies, and the authors did not respond to a request for clarification. Finally, for the paper by Aboyans et al., [13] the type of ICC calculated was not reported, and while pooling of this data would be possible, understanding which ICC was used is preferred to allow for accurate and appropriate calculation of the standard error. As there were only a small number of eligible papers identified we would require data from all articles to allow for appropriate pooling of ICCs. Thus, as a consequence of the small number of papers reviewed and insufficient data reported by several of the papers it was not possible to conduct a meta-analysis as part of this review. None of the authors responded to requests for missing data. A narrative review of results is presented instead.

### Inter-rater reliability

Inter-rater reliability results are included in Table 2. Statistical methods for calculating reliability were inconsistent. Of the eleven included studies, five reported levels of agreement with ICCs [13, 16, 46, 56, 57]. Of these, only three [13, 46, 56] reported 95% confidence intervals, which limits the interpretation of reliability in the context of clinically meaningful results. Based on ICC values alone, inter-rater reliability was highly variable, ranging from poor (ICC: 0.42) [16], to excellent (ICC: 1.0) [46].

Other estimates of reliability reported in included studies were coefficient of variation between raters [12, 49] (ranging from 3.2 to 5.9%), inter-observer reliability of 10% for raters [48], and a moderate Pearson’s correlation coefficient of 0.52 in a population with suspected PAD [50].

Of the remaining studies, one demonstrated statistically significant differences in ABI between raters in a population with severe PAD and in those with no disease, which did not occur in those participants with mild to moderate PAD [47], suggesting increased reliability with this disease state. In contrast, another paper reported Kappa coefficients of 0.4 (low agreement) for healthy limbs, 0.7 (good agreement) for limbs with PAD, and 0.43 (moderate agreement) for limbs with medial arterial calcification (MAC) (*p* < 0.001 for all values) [51].

### Intra-rater reliability

Intra-rater reliability results are included in Table 2. Various methods of calculating reliability were used. Of the eight included studies, four reported ICCs [13, 52, 56, 57], with ICC values ranging from poor (ICC: 0.42) [56] to excellent (0.98) [57]. Interpretation of the results was limited again by the fact that not all studies reported 95% confidence intervals, with only two articles having done so. [13, 56]. Other estimates of reliability included coefficient of variation [53–55] (range 4.95% [54] – 15.8% [55]), and an intra-observer variance of 8% [16].

## Discussion

The findings of this review are that the inter- and intra- tester reliability of the ABI across a number of mixed populations appears to be acceptable, however statistical tests of reliability in included papers were heterogeneous and levels of statistical reporting were inconsistent and incomplete. This makes interpretation of the reliability of the ABI in the context of clinical detection, evaluation and ongoing monitoring of peripheral arterial supply challenging, and prevented meta-analysis. For example, where studies lack 95% confidence intervals for ICCs, the validity of interpretation of the value is reduced as it fails to provide the lowest level of reliability that it represents. Similarly for coefficient or estimate of variation, values between 3.2 and 15.8% were reported.. Whilst this is considered an acceptable level of variation for many clinical tests, for the ABI it can represent a range of values that may indicate both normal and pathological results; which could reduce the ability of ABI to reliably determine the presence and extent of PAD. For example, assuming a variation of 15%, an ABI of 1.0 (which is considered ‘borderline’ when ABI is used as a screening tool [6]) could represent a true value between 0.85 (indicative of PAD) and 1.15 (‘normal’).

Further complicating the interpretation and generalisability of the inter- and intra-rater reliability results of included studies was the heterogeneity of participant populations. Whilst the majority of studies included older people with PAD risk factors or suspected PAD, three studies also included healthy participants [13, 16, 52], and two used an exclusively young and healthy population [46, 56]. In clinical practice, ABI is used to evaluate peripheral arterial supply in people with risk factors for atherosclerosis, and in those with clinical signs and symptoms of PAD. The variation in the disease status of participants across the studies included in this review provides some difficulty in evaluating how the studies’ findings apply to the people in whom the ABI would clinically be used. The study that reported near-perfect inter- and intra- tester reliability included only healthy individuals under the age of 30 [56]. This population would not typically undergo vascular screening, and the results obtained do not indicate the ability of the ABI to perform reliably in the presence of pathology where the result is likely to be lower and therefore change in result indicative of worsening pathology is likely to be small. In contrast, inter-tester and intra-tester reliability was found to be poor in several populations in which this test is recommended including people with diabetes and without MAC, [51] and older people with risk factors for PAD [16].

Methodological differences between studies is also likely to have contributed to variable reliability outcomes, with automated oscillometric devices demonstrating marginally better reliability than manual assessment using Doppler [49, 55], while Doppler evaluation was found to be more reliable than the use of pulse palpation [13] or stethoscope [46]. Higher ABI reliability was found in more experienced raters [47]. Whilst most of the studies reported that participants rested for 5–15 min prior to testing [12, 13, 16, 46, 48, 51, 55–57], six studies did not describe any pre-test preparation [47, 49, 50, 52–54], and only one paper took steps to ensure that participants did not consume alcohol, caffeine or tobacco (which are known to affect blood pressure) in the two hours prior to testing which may have affected measurements, particularly when taken across two different testing sessions [55]. This lack of reporting of the methodology used to obtain systolic blood pressure measurements makes it difficult to compare results across the included studies as it is unknown how much external factors are likely to contribute measurement variability.

Two papers identified the presence of diabetes mellitus as a factor that may affect reliability of the ABI [12, 51], however only one study included a large enough sample of this cohort to perform statistical tests [51]. This study, which used only participants with diabetes, reported the Kappa coefficient for inter-tester measures for participants classed as having PAD or not, rather than performing ICCs on the measures obtained. The authors reported ‘good’ reproducibility of the ABI (Κ 0.7) in people classified by their ABI measurement as having PAD, but low reproducibility in those without PAD and in those with MAC. Previous research has also shown that people with diabetes demonstrate a different response to pre-measurement rest [22], and that brachial blood pressure measurement is also less reliable in these individuals [58]. Diabetes-related autonomic neuropathy has been shown to affect blood pressure regulation, with a lack of vasoconstriction arising from reduced sympathetic input, particularly in response to changes in temperature and position [59, 60].

### Limitations

While the search methods employed in this study were designed to be robust, there may be some evidence that was not captured, for example unpublished data. Further limitations to this study are the inability to perform meta-analysis in order to obtain a quantitative analysis of the available reliability data for the ABI, and the inability perform any sub-analyses relating to individual populations such as those with diabetes, or methods of measurement such as automated or manual methods. Furthermore, there has been some disagreement in the literature about which pressure measurement should be used to calculate the ABI [61, 62], with no studies exploring the effect of calculation method on reliability. However, the method of calculation cannot be excluded as a factor affecting reliability that has not been considered by this review.

## Conclusion

Results of included studies suggest the inter- and intra-tester reliability of the ABI is acceptable. However, inconsistencies in obtaining systolic pressure measurements, calculating ABI values, and incomplete reporting of methodologies and statistical analysis make it difficult to determine the validity of the results of included studies. Further research of ABI reliability using a more consistent approach to study design and implementation and more detailed reporting of results in populations with vascular pathology and at risk of PAD is required. Based on current available data clinicians should ensure they interpret ABI results in the context of other vascular assessment findings, and patient management is not based upon this measurement alone.

## Abbreviations

ABI: ankle brachial index
IC: intermittent claudication
ICC: intraclass correlation coefficient
MAC: medial arterial calcification
PAD: peripheral arterial disease
QAREL: Quality Appraisal of Reliability
